# Cryotherapy for Prevention of Taxane-Induced Peripheral Neuropathy: A Meta-Analysis

**DOI:** 10.3389/fonc.2021.781812

**Published:** 2021-11-29

**Authors:** Junting Jia, Yimeng Guo, Raghav Sundar, Aishwarya Bandla, Zhiying Hao

**Affiliations:** ^1^ Department of Pharmacy, the Affiliated Tumor Hospital of Shanxi Medical University, Taiyuan, China; ^2^ Department of Haematology-Oncology, National University Cancer Institute, National University Hospital, Singapore, Singapore; ^3^ The N.1 Institute for Health, National University of Singapore, Singapore, Singapore; ^4^ Yong Loo Lin School of Medicine, National University of Singapore, Singapore, Singapore; ^5^ Cancer and Stem Cell Biology Program, Duke-NUS Medical School, Singapore, Singapore; ^6^ National University Cancer Institute, National University Hospital, Singapore, Singapore

**Keywords:** cryotherapy, prevention, taxane, peripheral neuropathy, meta-analysis

## Abstract

**Purpose:**

Taxanes are widely used in gynecological cancer therapy, however, taxane-induced peripheral neuropathy (TIPN) limits chemotherapy dose and reduces patients’ quality of life. As a safe and convenient intervention, cryotherapy has been recommended as a promising intervention in the recent clinical guidelines for the prevention of TIPN. Although there are a considerable number of studies which explored the use of cryotherapy in preventing chemotherapy-induced peripheral neuropathy (CIPN), there is insufficient large-scale clinical evidence. We performed a meta-analysis on the current available evidence to examine whether cryotherapy can prevent TIPN in cancer patients receiving taxanes.

**Methods:**

We searched databases including PubMed, Embase, and Cochrane from inception to August 3, 2021 for eligible trials. Clinical trials that examined the efficacy of cryotherapy for prevention of TIPN were included. The primary outcome was the incidence of TIPN, and secondary outcomes were incidence of taxane dose reduction and changes in nerve conduction studies. The meta-analysis software (RevMan 5.3) was used to analyze the data.

**Results:**

We analyzed 2250 patients from 9 trials. Assessments using the Common Terminology Criteria for Adverse Events (CTCAE) score showed that cryotherapy could significantly reduce the incidence of motor and sensory neuropathy of grade≥2 (sensory: RR 0.65, 95%CI 0.56 to 0.75, *p*<0.00001; motor: RR 0.18, 95% CI [0.03, 0.94], *p*=0.04). When evaluated using the Patient Neuropathy Questionnaire (PNQ), cryotherapy demonstrated significant reduction in the incidence of sensory neuropathy (RR 0.11, 95% CI 0.04 to 0.31], *p*<0.0001), but did not show significant reduction in the incidence of motor neuropathy (RR 0.46, 95% CI 0.11 to 1.88, *p*=0.28). Cryotherapy was associated with reduced incidences of taxane dose reduction due to TIPN (RR 0.48, 95% CI [0.24, 0.95], *p*=0.04) and had potential to preserve motor nerves.

**Conclusions:**

Cryotherapy is likely to prevent TIPN in patients receiving taxanes. High quality and sufficient amount of evidence is warranted.

## 1 Introduction

Taxanes are extensively used as first-line chemotherapy agents in the treatment of many types of cancer including breast, ovarian, prostate, gastric, head-and-neck cancers, and non-small cell lung cancer. However, as one of the most common non-hematologic adverse effects, taxane-induced peripheral neuropathy (TIPN) has very high incidence ranging from 11 to 87% and severely impairs patients’ quality of life. TIPN primarily alters sensation in the extremities, with numbness, tingling, or pain in the fingertips and toes. It also presents as motor nerve impairments with significant weakness in the limbs, which may put patients at high risks of falling, or trouble sensing tiny objects (1, 2).

Due to the evolving knowledge base of the pathophysiology and diversity of mechanisms and risk factors, there have been no successfully proven medical treatments or managements to alleviate TIPN, and preventive treatments are urgently sought to reduce its incidence (3). There are no agents recommended for prevention of chemotherapy induced peripheral neuropathy (CIPN) in the clinical practice guidelines from the American Society of Clinical Oncology and the European Society for Medical Oncology (4, 5). Due to the lesser adverse effects, non-pharmacological interventions such as exercise, yoga, acupuncture and cryotherapy are being investigated for their role in prevention of CIPN in clinical trials, some resulting in reduced CIPN incidence to a certain extent (1).

Cryotherapy has been successfully used in reducing the burden of chemotherapy-related oral mucositis, alopecia, cutaneous toxicity, and onycholysis by decreasing regional perfusion with acceptable tolerability (6–9). In a study identifying risk factors of docetaxel-associated neuropathy, researchers found that patients wearing frozen gloves and socks showed lesser CIPN occurrence than those not using them (10). Following this report, several clinical trials investigated the effect of cryotherapy on preventing CIPN. However, there are no large-scale studies, and results remain inconclusive. Based on the characteristics of existing studies, we conducted a meta-analysis to compare and integrate the results of different studies, providing more objective data for applying cryotherapy to prevent TIPN in the future.

## 2 Methods

### 2.1 Literature Search

The Preferred Reporting Items for Systematic Reviews and Meta-Analyses (PRISMA) statement for reporting systematic reviews was followed (11, 12). Databases including PubMed, Embase, and Cochrane from inception to August 3, 2021 were searched using search terms: cryotherapy or cold therapy or hypothermia, and neuropathy, and taxane or paclitaxel or docetaxel or abraxane. Reference lists of all retrieved articles were manually searched for potentially relevant studies. We also corresponded with authors *via* email to seek any further information where required.

### 2.2 Study Selection

Initially included trials were manually screened from the searched articles based on titles and abstracts. Articles such as conference abstracts, case reports and reviews were excluded. Studies on animals, or written in languages other than English were also excluded. Finally, the full-text of studies were carefully assessed.

Studies evaluating the efficacy of cryotherapy in prevention of peripheral neuropathy in cancer patients receiving taxane chemotherapy and were included. Both randomized and non-randomized trials were included. We considered patients who had cancer and who received taxanes for chemotherapy. Patients who had already developed CIPN were excluded. Cryotherapy were administered in any mode of physical cooling treatment on feet and (or) hands administered along with chemotherapy. Cryotherapy was a prophylactic intervention against TIPN in patients, while studies that examined cryotherapy as a treatment of TIPN had developed in patients were excluded. The control groups such as no intervention with cryotherapy in different patients or in different limbs were accepted. Patients using cryotherapy as treatment were also accepted as control. Our primary outcome was the incidence of peripheral sensory neuropathy and peripheral motor neuropathy induced by taxane ≥grade 2 as evaluated by the Common Terminology Criteria for Adverse Events (CTCAE) or ≥grade D as evaluated by the Patient Neuropathy Questionnaire (PNQ) (13). The secondary outcomes included occurrence of dose reduction of chemotherapy (dose reduction or cessation of chemotherapy) due to TIPN, changes in nerve conduction study (NCS) parameters including sensory nerve action potential (SNAP) and compound motor action potential amplitudes (cMAP).

### 2.3 Data Extraction

Two reviewers independently extracted the following parameters from the included studies: first author, year of publication, study type, type of cancer, chemotherapy regimen, experiment and control arms, sample size, and outcomes of treatment. Discrepancies between the two reviewers were resolved by discussion and consensus.

### 2.4 Quality Assessments

The quality of the included randomized controlled trials (RCTs) was assessed using the modified Jadad scale which evaluates the study quality on a scale of seven based on their description of randomization, blinding, concealment of allocation and withdrawals (14).

Methodological index for nonrandomized studies (MINORS) with some modifications were applied to evaluate the quality of non-randomized controlled trials (nRCTs) (15). The following 12 items were evaluated for each study: a clearly-stated aim, consecutive patients, prospective data collection, reported endpoints, unbiased outcome evaluation, adequate length of follow-up, loss to follow-up <5%, ≥20 patients in each arm, adequate control group, contemporary groups, controls equivalent to cases, and adequate statistical analyses.

### 2.5 Statistical Analysis

Statistical analysis was carried out using Review Manager version 5.3 (The Cochrane Collaboration, Software Update, Oxford). Dichotomous variables were calculated by risk ratios (RR) with a 95% confidence interval (CI). Continuous variables were calculated by weighted mean difference with a 95% CI. Heterogeneity was evaluated with χ^2^ and *I* ^2^ statistics. If heterogeneity between studies was considered significant (I^2^>50%, *p*<0.1), data was analyzed using a random-effects model and sensitivity analysis was conducted to search source of heterogeneity; otherwise, a fixed-effects model was adopted (I^2^< 50%, *p*>0.1). Sensitivity analysis was performed by removing each study sequentially to detect its influence on the results of this meta-analysis. The overall effect was evaluated using Z scores, in which *p*<0.05 was considered statistically significant.

## 3 Results

### 3.1 Study Characteristics

**Figure 1:**
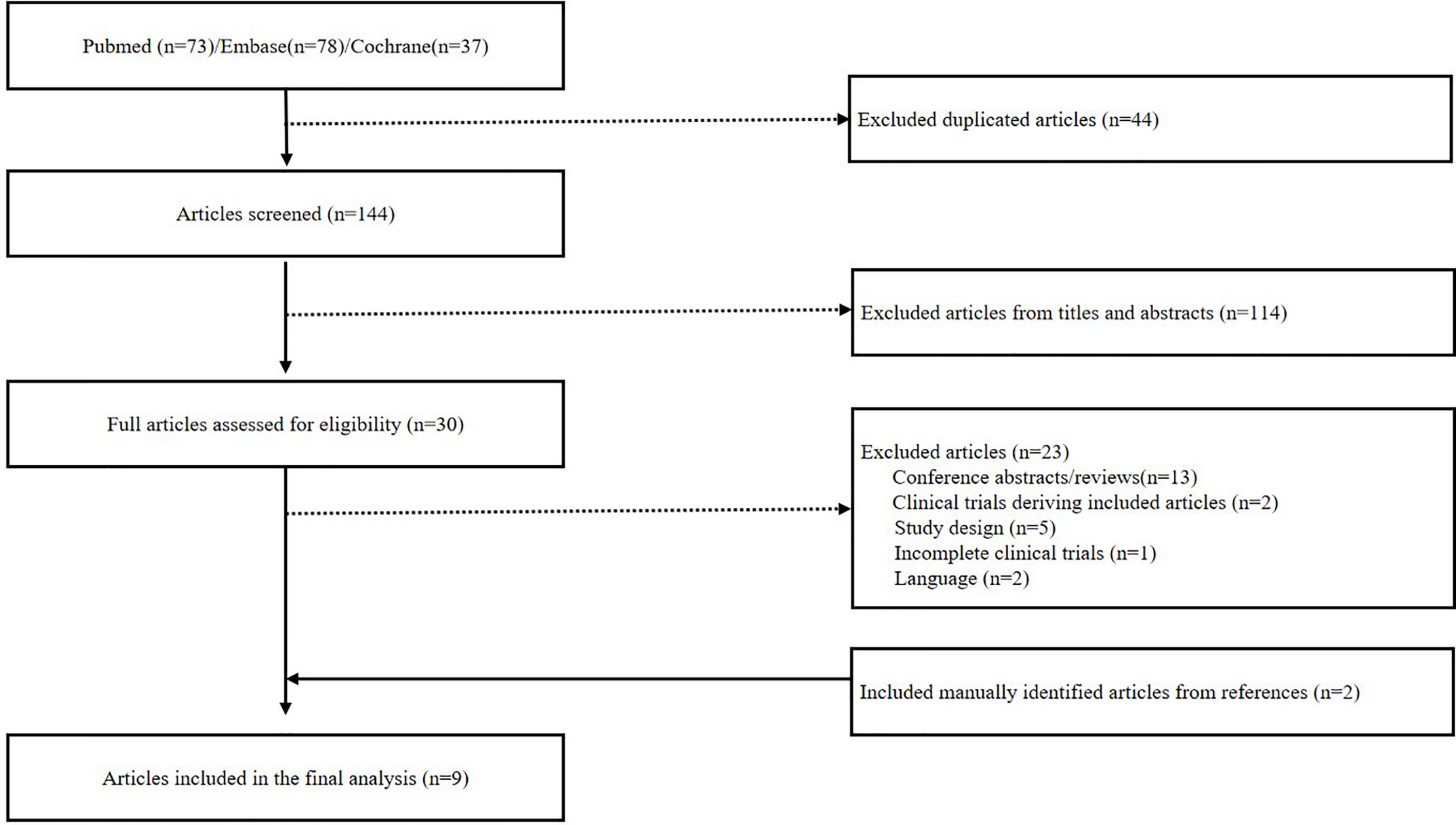
Flow chart of literature search and screen.

Eligible studies were screened following PRISMA flow diagram guidelines as shown in **Figure 1** (12). A total of 188 articles were initially identified from database search (**Figure 1**), from which 44 duplicate articles were removed. Then, 114 articles were excluded on screening for titles and abstracts. The remaining 30 articles were read carefully, among which 23 articles were excluded for following reasons, such as incomplete trials, clinical trials reporting studies already in the included articles, improper study design and conference abstracts/reviews. Additionally, two articles were identified from references of the full text of the above articles which met the eligibility criteria. Finally, nine studies were chosen for this analysis including three RCTs and six nRCTs.

Characteristics of the included studies are summarized in **Table 1**. Among these studies, Bandla et al. (19), Eckhoff et al. (10), Ng et al. (16), Rosenbaek et al. (21), Ruddy et al. (17), Sato et al. (22), Shigematsu et al. (18) and Shimanuki et al. (23) involved participants receiving cryotherapy measures in the intervention group, while the rest of the participants were set as the control group. Meanwhile, Hanai et al. (20) designed self-controlled studies with participants receiving cryotherapy measures as intervention on one hand/foot, and the other hand/foot as non-intervention control. Totally, outcomes of 36 participants were obtained from the self-controlled studies, while in the remaining eight studies, 946 patients were given prophylactic cryotherapy and 1268 were allocated as controls with no cryotherapy.

**Table 1:** Characteristics of the included trials.

Study (Year)	Study type	Cancer type/Chemotherapy regimen	Experimental/Control arm (No.)	Outcomes	Quality scores
Ng et al. (16)	RCT	Breast cancer/paclitaxel	Frozen gloves and socks on all extremities/no (17/21)	PNQ grade ≥D for sensory and motor peripheral neuropathy	5/7
Ruddy et al. (17)	RCT	Breast cancer/paclitaxel	Cryotherapy/standard therapy (21/21)	CTCAE grade ≥2 for sensory peripheral neuropathy	3/7
Shigematsu et al. (18)	RCT	Breast cancer/paclitaxel	Frozen gloves and socks/no (22/22)	CTCAE grade ≥2 and PNQ grade ≥D for sensory and motor peripheral neuropathy	4/7
Bandla et al. (19)	nRCT	Breast cancer/paclitaxel; Prostate cancer/docetaxel	Four-limb cryocompression and continuous-flow cooling/control (34/21)	Nerve conduction studies	16/24
Eckhoff et al. (10)	nRCT	Breast Cancer/docetaxel	Frozen gloves and socks/no (686/862)	CTCAE grade ≥2 for sensory peripheral neuropathy	17/24
Hanai et al. (20)	nRCT	Breast cancer/paclitaxel	Frozen gloves and socks on the dominant side/untreated sides (36/36)	PNQ grade ≥D for sensory peripheral neuropathy	20/24
Rosenbaek et al. (21)	nRCT	Breast cancer/paclitaxel	Prophylactic cryotherapy/therapeutic cryotherapy (96/119)	Dose reduction due to TIPN	16/24
Sato et al. (22)	nRCT	Gynecologic cancer/paclitaxel	Regional cooling/control (40/142)	Dose reduction due to PN	16/24
Shimanuki et al. (23)	nRCT	Early-stage breast cancer/paclitaxel	Cryotherapy applied to both hands and feet/no cryotherapy (26/64)	CTCAE grade ≥2 for sensory peripheral neuropathy, dose reduction due to PN	21/24

### 3.2 Primary Outcomes

**Figure 2:**
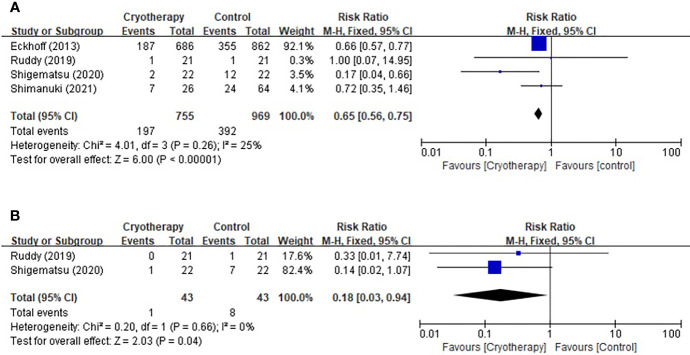
Forest plots of the preventive effects of cryotherapy on TIPN evaluated by CTCAE**≥**grade 2. **(A)** sensory neuropathy; **(B)** motor neuropathy.

Based on the CTCAE for TIPN, four studies included evaluation of peripheral sensory neuropathy and two trials included motor neuropathy assessment. As shown in **Figure 2**, cryotherapy was associated with significantly reduced incidence of grade≥2 TIPN-sensory (RR 0.65, 95% CI [0.56 to 0.75], *p*<0.00001). Meanwhile, cryotherapy also significantly reduce the incidence of grade≥2 TIPN-motor (RR 0.18, 95% CI [0.03, 0.94], *p*=0.04).

**Figure 3:**
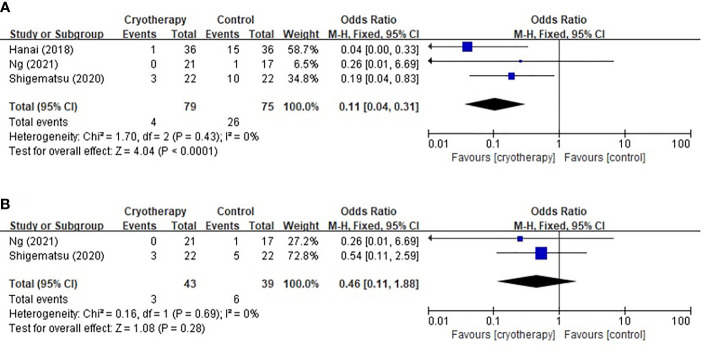
Forest plots of the preventive effects of cryotherapy on TIPN evaluated by PNQ≥grade D. **(A)** sensory neuropathy; **(B)** motor neuropathy.

Three trials using the PNQ assessment tool were included to analyze the effect of cryotherapy in preventing TIPN. As shown in **Figure 3**, it was concluded that while cryotherapy was associated with a significant reduction of incidence of grade≥D sensory neuropathy, however, it was not associated with significantly reduced incidence of grade≥D motor neuropathy (sensory: RR 0.11, 95% CI [0.04, 0.31], p<0.0001; motor: RR 0.46, 95% CI [0.11, 1.88], *p*=0.28).

### 3.3 Secondary Outcomes

#### 3.3.1 Occurrence of Chemotherapy Dose Reduction

**Figure 4:**
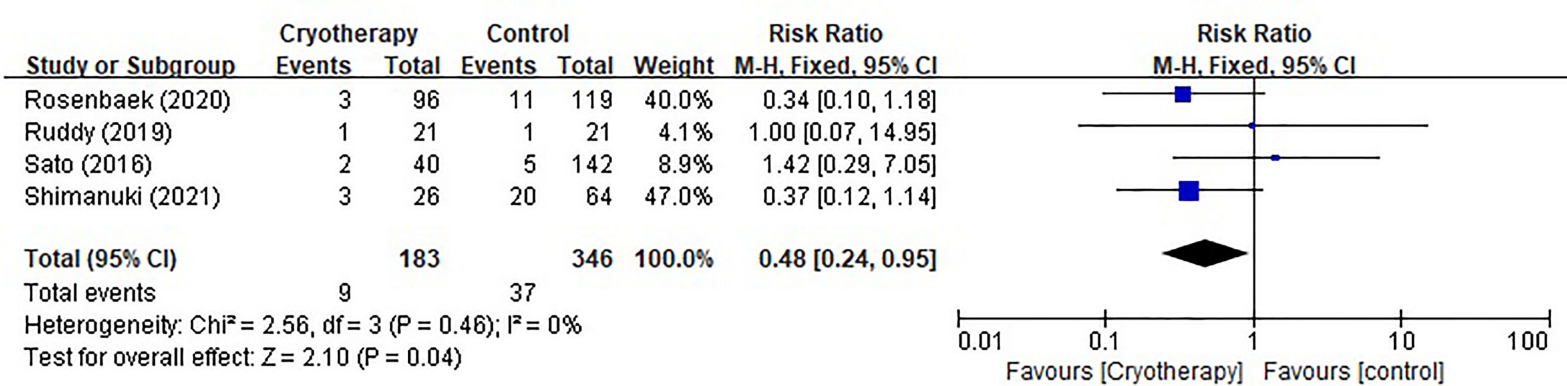
Forest plots of preventive effect of cryotherapy on incidence of taxane dose reduction for TIPN.

Four studies evaluating chemotherapy dose reduction due to TIPN were included as shown in **Figure 4**. The results indicated that incidence of taxane dose reduction was significantly decreased in patients receiving cryotherapy (RR 0.48, 95% CI [0.24, 0.95], *p*=0.04).

#### 3.3.2 Effects of Cryotherapy on Nerve Conduction Studies

Two studies focusing on the effects of cryotherapy on nerve conduction were included. In Bandla’s study (19), NCS was conducted at three time points-before the start of chemotherapy, after chemotherapy and 3 months after chemotherapy. While in Ng’s study (16), NCS was conducted at three time points-before the start of chemotherapy, 1-2 weeks and 6 months after chemotherapy. From the former study, subjects treated with cryotherapy showed significantly better preservation of cMAP amplitudes than control group, while cryotherapy did not significantly affect taxane-induced changes in SNAP amplitudes. From the later study, no difference was observed at all time points with sural SNAP or peroneal cMAP motor amplitudes.

### 3.4 Sensitivity Analysis of Studies With Significant Heterogeneity

Results of heterogeneity in outcomes of incidence of TIPN based on CTCAE and PNQ, occurrence of chemotherapy dose reduction due to TIPN were not significant. Thus, sensitivity analysis was not performed further.

## 4 Discussion

As a dose-limiting adverse effect, TIPN may start days after the first dose and tend to improve after stopping the treatment. In some patients, symptoms can continue up to 1-3 years after completion of the therapy and can sometimes last lifelong (24). Therefore, TIPN significantly reduces the living quality of cancer patients treated with taxanes. Cryotherapy was shown to prevent TIPN through local vasoconstriction in an animal study, further reducing delivery of the neurotoxic chemotherapy to the peripheral nerves (25). Moreover, cryotherapy is a safe and easy-to-operate method in clinical settings (26). Thus, cryotherapy is considered a promising method for TIPN. Based on the studies we reviewed, cryotherapy has potential to reduce the incidence of TIPN in terms of CTCAE≥2 on motor and sensory neuropathy (10, 17, 18, 23), and PNQ≥D on sensory neuropathy (16, 18, 20). It also decreased the incidence of chemotherapy dose reduction due to TIPN (17, 21–23) and preserved the motor nerve well based on cMAP at post-chemotherapy 3 months (19). However, there were no significant improvements in patients treated with cryotherapy on the incidence of TIPN in terms of PNQ≥D on motor neuropathy (16, 18). Sensory nerve had not been preserved well based on SNAP (19).

For the primary outcomes, a beneficial effect of cryotherapy on the incidence of taxane-induced sensory neuropathy was found, measured using both CTCAE and PNQ; yet conflicting results were seen relating to motor neuropathy. The possible explanation for these results was that the two measurements techniques have different sources (27): CTCAE is a physician-based instrument, while PNQ is patient-based. For sensory neuropathy, the judgement of physicians is mainly according to the dictation of patients based on their subjective feeling, meanwhile, PNQ is recorded by patients based on their own experiences. Thus, with similar sources, both CTCAE and PNQ for sensory lead to similar results. On the other hand, the observed discrepancy in motor neuropathy assessed with CTCAE and PNQ can be attributed to the reason that patients tend to be more aware of the CIPN symptoms in relation to how they impact their activities of daily living, while physicians may generally judge the absolute detectable level of muscle weakness to be of greater importance than symptom levels (13). Although CTCAE is widely used, the use of PNQ is more valuable in the clinical setting (13).

As shown in the included studies, hypothermia devices were mostly administered on hands and (or) feet of patients. For blood flow was reported to decrease by about 50% when the epidermal temperature fell to about 20°C (28), hypothermia locally may reduce the drug distribution in corresponding position. From the mechanism, cryotherapy could reduce the distribution of taxanes on hands and feet, further, decrease the aggregation and bundling of microtubule, restrain the changes in cell shape and cell stability and impairment of axonal transport of essential cellular components, and stop the degeneration of distal nerve segments and axonal membrane remodeling ultimately (24). From clinical aspects, sensory nerve abnormalities induced by taxanes like pain, numbness and tingling are most common and often occurs on hands and feet (24), cryotherapy on related parts may directly influence the sensory neuropathy. Thus, the significantly reduction of occurrence of sensory neuropathy with cryotherapy in our analysis can be explained.

Motor neuropathy induced by taxane is less common than sensory neuropathy, and includes a mild distal weakness associated with myalgia, especially of the toe extensor muscles (29). It was also reported that this paclitaxel acute pain syndrome occurs as a result of sensitization of nociceptors, their fibers or the spinothalamic system, as opposed to a musculoskeletal injury (24). From the mechanism and clinical aspects, it cannot be concluded that hypothermia has the potential to reduce the occurrence of motor neuropathy. In our analysis, the incidence of TIPN in terms of PNQ≥D on motor neuropathy was not significantly reduced by cryotherapy, which was more prone to be accepted than results from CTCAE≥2 as previous explanation.

A positive result was found in the efficacy of cryotherapy on the dose reduction of taxanes due to TIPN incidence, in this analysis. The ultimate patient-oriented aims associated with prophylaxis of chemotherapy-induced adverse effects are to avoid dose limitations, complete chemotherapy, and achieve disease control (30, 31). Thus, cryotherapy is likely to enhance the full dose completion of chemotherapy and enhance patient survival.

TIPN is a result of peripheral axonal damage, and thus conventional neurophysiological methods may provide complementary information to clinical assessments (3, 5). In our study, the reductions of SNAP and cMAP amplitudes were chosen as secondary outcomes, and cryotherapy showed protective effects on cMAP amplitudes. This result may be explained by the thought that in contrast to sensory parameters, cMAP amplitudes are dependent not only on nerve axonal function but additionally on muscle fiber function. Considering that taxane-induced motor neuropathy has been rarely reported, much of the cMAP change may in fact reflect taxane-induced myopathy which is infrequently reported due to poor methods of detection (32, 33). While cryotherapy seems to have a more pronounced positive effect on muscle parameters than nerve, the preservation of cMAPs could be an effect of reduced occurrence of myopathy (27, 34).

Our study had some strengths. Firstly, to our knowledge, this is the first meta-analysis to have quantitatively examined the effects of cryotherapy on the prevention of TIPN. Although Bailey et al.’s (35) study systematically reviewed the preventive effect of cryotherapy on TIPN and discussed the contradicting evidence, it was not analyzed further. Secondly, most of former meta-analyses about treatments of CIPN did not specify the chemotherapy regimens, which have different characteristics (13, 36, 37), while, in our study, only peripheral neuropathy induced by taxanes was analyzed and from which the results may be more reliable. Thirdly, although physical therapies (including touch therapy and acupuncture), dietary supplements (like vitamin E, glutamine, acetyl-L-carnitine, alpha-lipoic acid and omega-3 fatty acids), and herbal medicine (goshajinkigan) have been proven to have certain validity in the prevention of CIPN in pre-clinical and clinical trials, convincing evidence is still lacking (1). As a safe, non-invasive, and easy-to-operate method, cryotherapy is valuable to be evaluated for prevention of TIPN, and our study found promising positive effects of cryotherapy.

Our study also had some limitations. One limitation of this meta-analysis is the limited number and poor quality of trials on this topic. Another limitation is the differences in coolant temperatures and methods used in cryotherapy in the included trials. In studies from (16, 18, 20, 21), cryotherapy was performed using Elasto-Gel™ hypothermia gloves and socks (−20 to −10°C) on both hands and feet in the intervention group, 15 min before the paclitaxel infusion until 15 min post infusion, for a total of 90 min. In the study from Sato et al., cryotherapy was also administered using Elasto-Gel™ hypothermia gloves and socks (precooled to −30°C before use), but the insulators were fitted on both hands and feet for 3 h under paclitaxel administration (22). In studies from Ruddy et al. and Eckhoff et al., cryotherapy was both administered 15min before the chemotherapy until 15 min post infusion, while in the former patients wore cotton gloves or socks over their hands or feet which were each inserted into the pocket of a quart-sized plastic bag 2/3 or 1/2 full of ice, in the latter frozen gloves and socks (precooled to −30°C before use) were offered to patients (10, 17). In Shimanuki et al.’s study, cryotherapy was self-administered by patients using various cooling goods and methods including ice packs, refrigerants, cold 500 mL bottle-covered towels, and ice gloves (23). In Bandla et al.’s study, cryocompression at 16°C and cyclic pressure (5–15 mmHg) was administered to patients, comprising a pre-cooling period (1h), continued with the taxane infusion and a post-cooling period (on average, 30 min after the end of the taxane infusion) (19). Therefore, more high-quality, large-scale, and uniform approach of cryotherapy studies on TIPN are warranted to arrive at more convincing results.

## 5 Conclusion

Prophylactic cryotherapy is likely to prevent TIPN in patients. However, no definite protocols for cryotherapy have been recommended for the intervention parameters and dosing such as the variety in temperature control measures of current studies. Additionally, due to the low quality and limited number of clinical trials on this topic, the efficiency of cryotherapy in preventing TIPN is still inconclusive. Therefore, more high-quality and well-designed trials with standardized protocols are needed.

## Data Availability Statement

The original contributions presented in the study are included in the article/supplementary material. Further inquiries can be directed to the corresponding authors.

## Author Contributions

ZH and JJ contributed to the study conception and design. Literature search and data analysis were conducted by JJ and YG. The first draft of the manuscript was written by JJ. AB and RS critically revised and reviewed this paper. All authors contributed to the article and approved the submitted version.

## Conflict of Interest

RS has received honoraria from Bristol-Myers Squibb, Lilly, Roche, Taiho, Astra Zeneca, DKSH and MSD; has advisory activity with Bristol-Myers Squibb, Merck, Eisai, Bayer, Taiho, Novartis, MSD and AstraZeneca; received research funding from MSD and Paxman Coolers; and has received travel grants from AstraZeneca, Eisai, Roche and Taiho Pharmaceutical. AB has received travel funding from Paxman Coolers.

The remaining authors declare that the research was conducted in the absence of any commercial or financial relationships that could be construed as a potential conflict of interest.

## Publisher’s Note

All claims expressed in this article are solely those of the authors and do not necessarily represent those of their affiliated organizations, or those of the publisher, the editors and the reviewers. Any product that may be evaluated in this article, or claim that may be made by its manufacturer, is not guaranteed or endorsed by the publisher.
